# Implications of the Intestinal Microbiota in Diagnosing the Progression of Diabetes and the Presence of Cardiovascular Complications

**DOI:** 10.1155/2018/5205126

**Published:** 2018-11-12

**Authors:** Alina Mihaela Leustean, Manuela Ciocoiu, Anca Sava, Claudia Florida Costea, Mariana Floria, Claudia Cristina Tarniceriu, Daniela Maria Tanase

**Affiliations:** ^1^Department of Gastroenterology, “Sf. Spiridon” County Clinical Emergency Hospital, “Grigore T. Popa” University of Medicine and Pharmacy, Iasi, Romania; ^2^Department of Pathophysiology, Faculty of Medicine, “Grigore T. Popa” University of Medicine and Pharmacy, Iasi, Romania; ^3^Department of Morpho-Functional Sciences I, Faculty of Medicine, “Grigore T. Popa” University of Medicine and Pharmacy, Iasi, Romania; ^4^Department of Ophthalmology, Faculty of Medicine, “Grigore T. Popa” University of Medicine and Pharmacy, Iasi, Romania; ^5^Department of Internal Medicine, “Grigore T. Popa” University of Medicine and Pharmacy, Iasi, Romania; ^6^3rd Internal Medicine Clinic, “Sf. Spiridon” County Clinical Emergency Hospital, Iasi, Romania; ^7^Department of Morpho-Functional Sciences I, Discipline of Anatomy, Faculty of Medicine, “Grigore T. Popa” University of Medicine and Pharmacy, Iasi, Romania

## Abstract

The prevalence of diabetes is steadily rising, and once it occurs, it can cause multiple complications with a negative impact on the whole organism. Complications of diabetes may be macrovascular: such as stroke and ischemic heart disease as well as peripheral vascular and microvascular diseases—retinopathy, nephropathy, and neuropathy. Key factors that cause cardiovascular disease in people with diabetes include hyperglycemia, dyslipidemia, obesity, insulin resistance, inflammation, hypertension, autonomic dysfunction, and decreased vascular response capacity. Microbes can be considered a complex endocrine system capable of ensuring the proper functioning of the body but are also responsible for the development of numerous pathologies (diabetes, coronary syndromes, peripheral arterial disease, neoplasia, Alzheimer's disease, and hepatic steatosis). Changes in the intestinal microbiota may influence the host's sensitivity to insulin, body weight, and lipid and carbohydrate metabolism. Dysbiosis causes activation of proinflammatory mechanisms, metabolic toxicity, and insulin resistance. Trimethylamine N-oxide (TMAO) is a microbial organic compound generated by the large intestine, and its concentration increases in the blood after ingestion of foods rich in L-carnitine and choline, such as red meat, eggs, and fish. The interest for TMAO in cardiometabolic research has recently emerged, given the preclinical evidence that reveals a link between TMAO, diabetes, and cardiovascular complications. Intestinal microbiota can be modulated by changing one's lifestyle but also by antibiotic, probiotic, prebiotic, and fecal transplantation. The purpose of this article is to highlight issues related to the involvement of microbiota and trimethylamine N-oxide in the pathogenesis of diabetes mellitus and cardiovascular disease. Better appreciation of the interactions between food intake and intestinal floral-mediated metabolism can provide clinical insights into the definition of individuals with diabetic risk and cardiometabolic disease as well as potential therapeutic targets for reducing the risk of progression of the disease.

## 1. Definition

Microbiota is part of a complex system that includes all microorganisms, cells, antimicrobial peptides, luminous compounds, and all interactions between them [1, 2].

The microbiota is involved in numerous activities such as vitamin production, regulation of gene expression, fight against pathogenic bacteria, absorption of nutrients, and regulation of metabolic disorders [3].

Intestinal microbiota is influenced by factors such as genetics, lifestyle, diet, and antibiotherapy [2, 4, 5].

Microorganisms such as bacteria, viruses, and fungi survive in the gastrointestinal tract. The intestinal microbiota is the result of the complex interaction between the environment and host genetics; diet is the component that modulates the intestinal bacterial activity. An imbalance of intestinal homeostasis causes the internal dispersion of bacterial fragments and promotes intestinal permeability and bacterial and circulating endotoxin translocation, which initiates inflammation in tissues responsible for insulin metabolism thus causing insulin resistance [1, 6]. Dysbiosis also plays an important role in the pathogenesis of cardiovascular and metabolic diseases [7, 8].

The intestinal microbiota can be considered a gate that modulates the transition into cardiometabolic diseases that involve the hepatobiliary tract [4, 7].

## 2. Microbiome Composition

Our body is colonized by a series of microbiota, primarily bacteria, which exist in a symbiotic relationship with the host and play an important role in maintaining the homeostasis of the host [8]. The intestinal microbe is comprised of billions of cells, of which the most important are Gram-positive bacteria belonging to the phyla Firmicutes and Actinobacteria and also to the genera Clostridium, Bifidobacterium, Lactobacillus, Ruminococcus, and Streptococcus and Gram-negative bacteria belonging to the genera Bacteroides, Prevotella, and Akkermansia [2].

The link between microbiota compounds and the host's immune system is supported by a series of molecules and signaling processes that can affect the intestine, liver, brain, and other organs [9]. On the other hand, the intestinal immune system plays a significant role in the exposure of bacteria to host tissues, causing stratification of intestinal bacteria on the lumbar side of the epithelial barrier, and controls the composition of the intestinal microbiota [4]. Residual bacteria provide signals that favor the development of a normal immune system and regulate the resulting immune responses. Changing these can cause significant repercussions on the host's health [9]. The disruption of the intestinal microbe may also be associated with numerous pathologies: metabolic syndrome, obesity, diabetes, renal disease, cardiovascular disease, neoplasia, and Alzheimer's disease [2]. Clostridium species correlate negatively with glucose, HbA1c, and insulin levels, whereas Lactobacillus species correlate positively with glucose and HbA1c levels [10]. It has been demonstrated that a higher blood glucose concentration can be predicted by reducing the proportion of anaerobes, especially Bacteroides [11]. Markers of glucose metabolism disorders (e.g., insulin and insulin resistance-HOMA-IR) are commonly associated with the microbial genotype, suggesting that people with fewer genotypes are predisposed to metabolic disorders and secondarily to diabetes [11].

**Figure 1:**
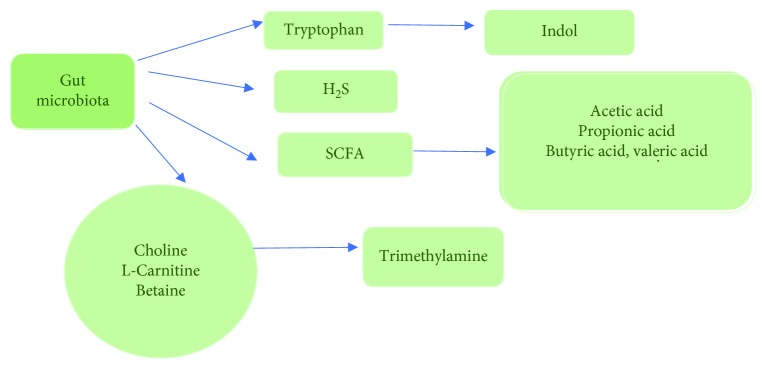
Gut microbiota metabolites.

The intestinal microbiota influences the host's health by the intestinal immune response. L-Tryptophan plays a significant role in maintaining the balance between intestinal microfibres and immune tolerance. Modification of the tryptophan metabolism influences the intestinal microbiota. Bacterial metabolites (indole, indolic acid, and tryptamine) and endogenes (serotonin, melatonin, and kynurine) influence the microbial metabolism, microbiota composition, and host immune system [9] (Figure 1).

Indoxyl sulfate is a metabolite of tryptophan-derived intestinal bacteria. Several studies show that indoxyl can affect the functions of the circulatory system by lowering NO (nitric oxide) production, increasing the production of reactive oxygen species, and promoting cardiac interstitial fibrosis [12].

Indole is a tryptophan metabolite that can regulate bacterial motility, antibiotic resistance, virulence, and intestinal biofilm formation. Indole catabolism is supported by tryptophanase, which can be induced by tryptophan or suppressed by glucose. Bacterial species, including *E. coli*, Proteus vulgaris, Paracolobactrum coliforme, Achromobacter liquefaciens, and Bacteroides spp., are capable of producing indole [9].

On the other hand, intestinal microbiota can use nutrients such as tryptophan, an essential amino acid, thereby reducing the supply of substrates for the endogenous synthesis of vital host compounds [13].

Indole administration can alleviate gastrointestinal tract damage induced by nonsteroidal anti-inflammatory drugs (NSAIDs), modulating inflammation mediated by innate immune responses and changes in intestinal microbial composition [9]. It has been shown that indole promotes the functions of the intestinal cell epithelial barrier by fortifying tight epithelial junctions between cells via receptor X (PXR), which may contribute to inflammation resistance. Indole can also enhance glucagon-like peptide-1 (GLP-1) secretion, an incretin with profound influences on host metabolism [4, 9].

For normal cells, exposure to indole can strengthen the mucosal barrier and mucin production by inducing expression of associated genes, thus increasing resistance to pathogenic invasion. For inflammatory cells, indole exposure can suppress the activation of NF-*κ*B chemokine production and, at the same time, increase the production of anti-inflammatory cytokines, thus improving inflammation and damage [9].

Most of the compounds derived from the intestine first enter the liver; systemic effects can also be exerted by hepatic metabolites of compounds derived from intestinal bacteria or changes in liver metabolism. To access the circulation, molecules derived from intestinal bacteria have to cross the intestinal barrier (GBB) [10, 13]. Some clinical and experimental studies show that cardiovascular disease may affect GBB function and that GBB permeability may be a new marker in CVD. In this case, a key factor for the proper functioning of GBB is proper blood perfusion through the intestines [13].

Multiple studies suggest that intestinal microbiota could produce biologically active compounds that enter the circulation and affect circulatory system homeostasis. To enter the circulation, intestinal bacterial metabolites have to pass through the intestinal barrier (GBB). The integrity and permeability of the intestinal barrier depend on numerous factors, including intestinal blood flow [14].

The intestinal blood barrier is made up of several layers. The internal or mucosal layer prevents pathogens from adhering to epithelial cells. The physical barrier consists of a single layer of enterocytes connected by tight junctions, which play a crucial role in the selectivity of the intestinal barrier [15].

Intestinal bacteria produce many vital nutrients for human homeostasis, such as vitamins K and B and SCFA which contribute to the transformation and degradation of biliary acids and steroids [13].

Intestinal metabolites such as hydrogen sulfide (H2S), SCFA, indole, or trimethylamine may exert effects on circulatory system homeostasis and on nerve and humoral control. Short-chain fatty acids, including acetic, propionic, butyric, and valeric acids, are formed from carbohydrates by bacterial fermentation [14] (Figure 1). The amount of SCFA may have vasorelaxant effects on the arterial resistance in the colon, improving microcirculation. SCFA depends on the composition of intestinal bacteria, diet, and intestinal transit time and plays a local role as an energy source for intestinal cells suppressing the growth of pathogens by reducing the pH in the intestines. There is also some evidence that SCFA derived from intestinal bacteria can affect blood pressure [13].

Short-chain fatty acids (SCFA), produced by bacterial fermentation in the colon, contribute to a significant proportion of the daily energy requirement. SCFA, especially butyrate and propionate, play an important role in differentiating regulatory T cells and regulating immunity in the intestinal tract. Increased production of acetate by intestinal microbiota could lead to the activation of the parasympathetic nervous system, which promotes increased secretion of insulin stimulated by glucose, hyperphagia, and obesity [16].

Intestinal bacteria produce many biologically active molecules, some of which play a role in regulating the circulatory system and energy balance. Besides numerous metabolites, intestinal flora produces methylamine, including TMA, dimethylamine, and monomethylamine. The intestinal barrier is considered a functional, immunological, and anatomical unit, separating the intestinal lumen from circulating blood, preventing bacterial adherence and transport regulation. Methanolamine and other intestinal metabolites reach almost all tissues as small molecules thus affecting both neurohormonal and peripheral regulatory mechanisms [17].

A fat-rich diet causes bowel dysbiosis and reduces intestinal integrity. Recent studies have shown that dysbiosis may contribute to the development of inflammation and subsequently the progression of cardiovascular disease (CVD) by promoting two major risk factors—arterial hypertension and atherosclerosis [7, 10].

TMAO precursors such as choline, L-carnitine, *γ*-butyrobetaine, phosphatidylcholine, betaine, glycerophosphine, and crotonobetaine are metabolized by the intestinal microbiota to produce trimethylamine which is then further metabolized to TMAO by the monooxygenase 3 enzyme (FMO3) [18]. Metabolizing TMAO by FMO3 was linked to insulin sensitivity and glucose metabolism [19]. Several studies have also suggested a significant role of choline in regulating insulin resistance and glucose metabolism. Diet significantly affects the intestinal microbiota and the production of TMAO [20].

FMO3 (flavin monooxygenase 3) is the preponderant enzyme in the liver, and flavin monooxygenase 1 and flavin monooxygenase 2 (FMO1 and FMO2, respectively) can also cause TMAO oxidation. In some patients with FMO3 gene mutation, accumulation of trimethylamine (TMA) spreads in the body and is released through sweating and breathing, resulting in fish smell syndrome, a genetic disease [20].

The plasma level of TMAO in the human organism is in the range of 0.5-10 *μ*mol/L. Recently, a number of clinical trials have indicated a possible positive correlation between increased plasma TMAO and an increased risk of cardiovascular disease [13].

Choline is an essential nutrient which is both synthesized endogenously and obtained from various animal and plant products [21, 22]. Food-derived choline is generally metabolized in the liver and is involved in various biological processes, synthesis of acetylcholine neurotransmitter, lipoprotein, and membrane phospholipids [23]. Betaine is the direct oxidation product of choline, which is a metal donor in homocysteine remethylation, and plays an important role in maintaining stability and cellular volume [22]. Together, choline and betaine have been recognized as achieving hepatoprotection and improving insulin resistance [23].

The intestinal microbiota through enzyme activity can turn choline into trimethylamine (TMA), a harmful metabolite known for its strong ammonia smell. TMA is absorbed and transmitted to the liver, where it can be rapidly detoxified by monooxygenase 3 (FMO3) to be transformed into trimethylamine N-oxide (TMAO) [23].

Significant conversion of choline into TMA by the intestinal microbiota may, however, reduce the bioavailability of choline, which could affect the secretion of very-low-density lipoproteins, with increased accumulation of triglycerides in the liver and could stimulate hepatic steatosis [22–24].

Blood TMAO levels depend on many factors, including diet, intestinal barrier permeability, liver enzyme activity and methylamine excretion rate, composition, and activity of the intestinal microfibres [25]. Changes in intestinal microbiota may influence the host's sensitivity to insulin with the onset of diabetes. Several studies have shown that the level of TMAO is significantly associated with the risk of type 2 diabetes [26, 27].

Thus, FMO3 is suppressed by insulin, and also FMO3 levels are elevated by glucagon secretion from pancreatic *α* cells to stimulate an increase in blood sugar [28]. Both glucagon suppression and insulin resistance are correlated with weight loss and act together to improve glucose homeostasis following a dietary restriction with body mass loss [26, 29]. A high-fat diet has led to changes in the intestinal microbial composition causing insulin resistance [30].

TMAO may cause inflammation of the adipose tissue with disruption to the insulin signaling pathway. This mechanism plays an important role in the emergence of insulin resistance and subsequently in the evolution towards diabetes [31–33]. The increase in TMAO levels may result from dietary differences; intestinal microbiota plays an important role in varying TMAO levels. L-Carnitine is essential for the mitochondrial metabolism of long-chain fatty acids, and some studies have provided evidence of glycemic control and plasma lipid control following L-carnitine administration in type 2 diabetes [29]. However, other studies demonstrate the increased risk of diabetic complications in patients with higher circulating L-carnitine concentrations. The role of L-carnitine in cardiovascular health was recognized after discovering the proatherogenic nature of TMAO and its relationship to L-carnitine metabolism [26].

TMAO produces a proatherogenic macrophage phenotype that affects the metabolism of cholesterol and sterol in macrophages, intestines, and liver. Studies have shown that diabetes and body mass index (BMI) are associated with higher levels of TMAO [27, 28]. After ingestion of phosphatidylcholine or L-carnitine, circulating TMAO levels increase in 4 to 8 hours and normalize after 24 hours depending on renal clearance [30]. Trimethylamine N-oxide (TMAO), which is derived from intestinal metabolite-derived metabolites, is possibly linked to diabetic, atherosclerotic, and cardiovascular risk [25]. The circulating TMAO levels are elevated and associated with the severity of the disease and with patients with atherosclerosis, chronic kidney disease, and peripheral arterial disease [7, 8]. Previous studies have shown that the bacterial species belonging to the families Clostridiaceae and Peptostreptococcaceae have been associated with increased blood levels of TMAO in humans [8].

TMAO generates atherosclerosis, perhaps by forming foam cells in the arterial wall. High levels of TMAO affect lipid metabolism, and inflammatory response promotes endothelial dysfunction and exacerbation of platelet reactivation and stimulates thrombosis. This highlights the importance of this molecule for cardiovascular complications [28].

**Figure 2:**
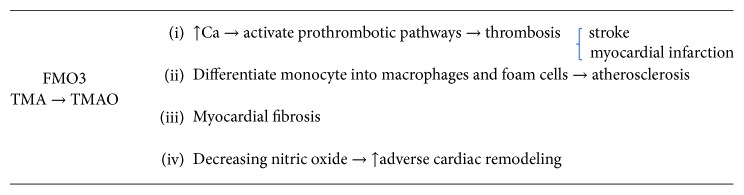
Effect of TMAO on cardiovascular disease.

TMAO activates in vascular smooth muscle cells and endothelial cells MKKK (mitogen-activated protein kinases), and the isolation of nuclear factor-*κ*B (NF-*κ*B) leads to increased expression of inflammatory genes and adhesion of endothelial cells to leukocytes. TMAO in vivo can increase the receptor expressed on CD36 and SR-A1, leading to the formation of foam cells, by a greater absorption of modified macrophage LDL. Furthermore, TMAO increases calcium concentration in the endoplasmic reticulum in the platelets, which consequently leads to platelet aggregation and thrombosis with increased risk of acute coronary syndromes [20] (Figure 2).

TMAO activates prothrombotic pathways by rapidly increasing the release of calcium ions (Ca^2+^) resulting in the activation of certain platelet stimuli. In endothelial cells and smooth muscle cells, TMAO rapidly activates the mitogen-activated protein kinase and the activated B cell activated kappa amplifier factor, which in turn favors the expression of adhesion molecules such as E-selectin [20]. TMAO can regulate and differentiate monocytes into foam cells and macrophages. TMAO can initiate profibrotic processes in the heart and kidneys by transforming the phospho-SMAD3 growth factor-*β* signaling axis [20, 34]. The association of all these complex cellular mechanisms accelerates atherosclerosis and thrombotic vascular disease and leads to secondary renal insufficiency [35]. Therefore, understanding the molecular mechanism of action of TMAO and discovering new mechanisms and receptors by which TMAO may lead to its adverse effects will have a much wider implication in clarifying its role in pathogenesis in humans [20, 35].

Increased levels of TMAO and choline are associated with low levels of HDL-cholesterol and plasma phospholipids [29]. Increased concentrations of TMAO in the blood influence the activity of intestinal microbiota and the permeability of the intestinal barrier and determine the activity of liver enzymes. There was a direct relationship between plasma TMAO concentrations, diabetes mellitus, acute coronary syndromes, and peripheral vascular disease [25, 36].

Furthermore, some experimental studies show that TMAO should affect lipid and hormone homeostasis, providing indirect evidence for the possible contribution of TMAO to the development of CVD. It has been found that TMAO could lead to decreased beta-oxidation of fatty acids through cardiac muscle cells. In addition to TMA, other metabolites have also been reported to play a role in the pathology of many diseases [16].

Indoxyl sulfate is produced by intestinal microbial tryptophanases that convert food tryptophan to indole, which is then transformed into indoxyl and indoxyl sulfate in the liver. It has been shown that indoxyl sulfate may have proinflammatory and prooxidant effects on cardiomyocytes and cardiac fibroblasts [16].

It is believed that complex molecules mediate intracellular and extracellular signaling, but it also appears that gaseous molecules, later referred to as “gas transmitters,” play an essential role in maintaining the body's homeostasis. The gas transmitters include carbon monoxide (CO), hydrogen sulfide (HS), and nitric oxide (NO), the cytotoxic molecule produced by phagocytic leukocytes [37].

The intestinal microbe uses sulfur-containing compounds to produce hydrogen sulfide. Hydrogen sulfide is an important biological mediator that is involved in various physiological processes, including blood pressure regulation [13, 16]. Moreover, phenylacetylglutamine is a product that is formed by the conjugation of phenylacetate and glutamine. Increased serum concentrations of phenylacetylglutamine should be a strong and independent risk factor for overall mortality and cardiovascular disease. Further studies are needed to elucidate the causal relationship between these metabolites and CVD [16].

## 3. Diabetes, Cardiovascular Diseases, and Microbiota

When the pancreas does not produce enough insulin or when the body cannot use insulin produced by the pancreas, diabetes can occur [38].

Diabetes mellitus is associated with an alteration of interdependent metabolic pathways (phospholipids, lipids, and methylation) and also with diseases such as retinopathy, nephropathy, neuropathy, and heart failure [36, 39].

Diabetes mellitus is a major risk factor for cardiovascular disease (CVD), which is the most common cause of death among adults with diabetes. The link between hyperglycemic status and microvascular disease is much more common than the link between hyperglycemic status and macrovascular disease, with a 37% increase in risk of renal failure or retinopathy [40].

Prevalence of diabetes increases with age. Diabetes patients are at a higher risk of developing cardiovascular disease. Inflammation and oxidative stress have a role in the mechanisms underlying cardiovascular disease and other complications in the development of diabetes [41]. Diabetics have a two to four times higher risk of developing cardiovascular complications and premature death. Myocardial ischemia is frequently asymptomatic in patients with diabetes and is associated with an unfavorable prognosis [42].

Diabetes causes various microvascular complications, such as autonomic and peripheral neuropathy, nephropathy, and retinopathy, and these complications are correlated with adverse cardiovascular effects [43].

Atherosclerosis of the large arteries and coronary arteries leads to macrovascular complications such as stroke, ischemic heart disease, and peripheral vascular disease. Atherosclerosis of small arteries causes diabetic nephropathy and is related to cardiovascular morbidity. Diabetes, regardless of its effect on atherosclerosis, is associated with changes in cardiac structure and function leading to myocardial dysfunction, called “metabolic cardiomyopathy” [42].

The presence of a metabolic syndrome exposes patients to an increased risk of cardiovascular complications. Moreover, in addition to classical risk factors, there are other unconventional factors that cause vasoconstriction and thrombosis, endothelial dysfunction, inflammation, oxidative stress, and vascular wall abnormalities that also contribute to an increased cardiovascular risk [40]. When the glycemic level falls below 70 mg/dL, autonomic nerve activation occurs. This may produce symptoms such as tremor, tachycardia, diaphoresis, anxiety, hunger, and headache [39, 40]. There are several mechanisms through which hypoglycemia could promote adverse cardiovascular effects in high-risk individuals. Hemodynamic changes following autonomous self-induced hypoglycemia include increased systolic blood pressure, heart rate, myocardial contractility, and cardiac output [36].

These effects can exacerbate ischemia. Hypoglycemia, as a complication of long-term diabetes, has also been associated with a prolonged QT interval. The relationship between hypoglycemia, autonomic neuropathy, and cardiac repolarization may contribute to arrhythmias and the risk of sudden death in people with diabetes [38]. Finally, hypoglycemia may have adverse effects on endothelial function, platelet reactivation, and coagulation cascade, and this increases blood viscosity and decreases serum potassium levels [40].

Concerns about cardiovascular complications associated with type 2 diabetes have traditionally focused on atherosclerotic vasculo-occlusive events such as myocardial infarction, stroke, and limb ischemia [41]. However, one of the most common and most serious cardiovascular disorders in patients with diabetes is cardiac insufficiency. Cardiac insufficiency and diabetes are physiologically related [42, 43]. Type 2 diabetes and heart failure have a common insulin resistance characteristic and are accompanied by the activation of the cascade of neurohormonal systems: norepinephrine, angiotensin II, aldosterone, and neprilysin. The two diseases overlap; diabetes is present in a proportion of 35-45% of patients with chronic heart failure, regardless of whether they have a reduced or conserved ejection fraction [44].

Reduced blood flow from the intestinal endothelium in patients with heart failure is due to a decreased cardiac output which causes intestinal wall ischemia, leading to disruption of the intestinal barrier function, increasing permeability [40, 43].

Systemic congestion in patients with heart failure may also cause edema of the intestinal wall, resulting in increased intestinal permeability. Thus, a translocation of endotoxins, microbial metabolites, and microbial components produced by Gram-negative bacteria entering the systemic circulation is determined at the intestinal level. These processes may further activate cytokines and may generate systemic inflammation that contributes to the progression of heart failure [7]. There is evidence of chronic heart failure (CHF) and gastrointestinal (GI) involvement in this syndrome. It is known that cytokine activation occurs in patients with chronic NYHA class III-IV heart failure, with both clinical severity and prognosis. It has been suggested that endotoxin may be an important stimulant for the production of cytokines in patients with chronic heart failure by its action on mononuclear cells [43–45]. According to this hypothesis, endotoxin enters the circulation through bacterial translocation in the intestine. The two main factors are intestinal edema and hypoperfusion. The finding that patients with edematous heart failure decompensation have elevated levels of endotoxin normalizing after diuretic treatment tends to suggest that edema of the intestinal wall may contribute to endotoxemia. In addition, there is evidence that intestinal hypoperfusion may result in mucosal ischemia, which may lead to increased intestinal wall permeability. Furthermore, the intestine may play an important role in the development of cardiac cachexia [45].

Bowel dysfunction and dysbiosis may contribute to metabolic disease. Diabetes mellitus and cardiovascular disease can compromise intestinal function by producing macro- and microangiopathy. In cardiac failure, there is centralization of circulation that further reduces intestinal perfusion. Intestinal ischemia leads to the progressive deterioration of the connections between enterocytes and an accelerated passage through the blood barrier [15].

Excessive intake of salt is considered a cardiovascular risk factor. Studies suggest that metabolites from intestinal bacteria, such as trimethylamine N-oxide (TMAO), are considered to be a potential marker of cardiovascular diseases and may affect homeostasis [12, 16]. Increased evidence suggests that homeostasis may very much depend on a mutualist relationship with intestinal bacteria and that CVD is associated with intestinal microbial dysbiosis. Research has shown that high salt intake is associated with increased TMAO in plasma and reduced urinary excretion of TMAO. Furthermore, it has been found that increased salt intake affects the intestinal microbial composition [12].

High blood pressure is a major risk factor for heart failure, coronary artery disease, and stroke, causing morbidity and high mortality. Hypertension is known to produce pathological changes in the vasculature, such as microangiopathy in the retina, kidneys, and other organs. However, there is insufficient data on the effect on hypertension in the intestinal vasculature [13, 14, 16].

A positive correlation between trimethylamine N-oxide in plasma at birth (TMAO) and the possible increased risk of major cardiovascular adverse events has been suggested; however, the value of diagnosing TMAO levels in blood in cardiovascular diseases is questionable. However, if the nutrient concentration exceeds the transport capacity of the small intestine, they reach the large intestine and are metabolized by intestinal bacteria that produce trimethylamine (TMA). Therefore, the concentration of TMAO in the blood may depend on a few factors, including diet, intestinal microbial activity, GBB permeability to TMA, liver and TMA oxidation, and TMA and TMAO excretion [14].

Dysbiosis can contribute to the evolution of high blood pressure, another risk factor for cardiovascular disease. Dysbiosis promotes hypertension by modifying vascular tone and developing vascular fibrosis [39]. Hypertension can be defined as reducing the arterial lumen with increased peripheral vascular resistance, resulting in increased blood pressure (BP). Also, intestinal dysbiosis contributes to hypertension by vasoconstriction induced by LDL oxidation [8].

High blood pressure is a major factor contributing to an increased risk of cardiovascular disease in patients with diabetes. The presence of hypertension in patients with type 2 diabetes increases the risk of myocardial infarction, stroke, and all-cause mortality. Combining both conditions increases the risk of heart failure, nephropathy, and other microvascular events [30, 40].

There is an association between diabetes mellitus and atrial fibrillation. Both have common precursors of hypertension, atherosclerosis, and obesity. Diabetes results from defects in insulin and glucose control. This, in turn, can directly affect the atrial and ventricular myocardium. The underlying mechanism of atrial fibrillation may be linked to inflammation, with high levels of C-reactive protein and also atrial fibrosis. Diabetes is also associated with the formation of proinflammatory mediators. Left ventricular hypertrophy (LVH) is a common consequence of high blood pressure, and both are recognized risk factors for atrial fibrillation. Multiple studies have shown an association with left ventricular hypertrophy and low glucose tolerance and insulin resistance [46].

Microbial intestinal changes have been linked to changes in insulin sensitivity and in glucose metabolism and the development of metabolic syndrome with diabetes and subsequent cardiovascular complications [26]. Reduced global microbial diversity in subjects with type 2 diabetes mellitus, diminution of Firmicutes bacteria (including Clostridia), and the occurrence of proteobacteria correlate with increased plasma glucose in the oral glucose tolerance test [47]. In diabetic patients, TMAO was found to be a significant marker for cardiovascular events. In addition, L-carnitine plasma concentrations in patients with high TMAO concentrations predicted an increased risk of cardiovascular disease and an increased incidence of major cardiac events [2, 6].

High plasma TMAO levels are associated with diastolic dysfunction and increased morbidity and mortality. TMAO may also cause ventricular remodeling by fibrosis, subsequent dilatation, thinning of the walls, and reduction of the ejection fraction [21]. Similarly, elevated levels of choline and betaine showed only an increased cardiovascular risk when associated with the concomitant increase in TMAO. These studies have enhanced the importance of dietary and antimicrobial therapy in cardiovascular health; TMAO level is a possible target for therapeutic interventions [27].

## 4. Treatment

Changing one's lifestyle can lead to a reduction in the risk of chronic illness, including obesity and diabetes [48].

Cardiovascular disease, the leading cause of death worldwide, poses an interest in investigation of intestinal microbiota as an interventional mechanism which yields new and clinically relevant information for future research and has a complex therapeutic potential [49].

Intervention on intestinal microbiota has already become a new target for both the prevention and treatment of complex cardiometabolic diseases [4].

The intestinal microbiota can be modulated by the administration of antibiotics, prebiotics, and probiotics or by fecal transplantation [26].

Prebiotics have protective effects and may reduce the risk of cardiovascular disease and diabetes by having a positive impact on the growth of beneficial microbial flora. Probiotics are new therapies for treating hypercholesterolemia [2, 50].

The administration of probiotics stimulates the immune response, improves lactose tolerance, has anti-inflammatory effect, and even regulates intestinal disorders caused by obesity [1, 49]. Probiotics are living nonpathogenic microorganisms that provide benefits to the host [1, 21, 43]. The intestinal microbiota may play an important role in the pathogenesis of type 2 diabetes, influencing body weight, proinflammatory activity, bile acid metabolism, insulin resistance, and intestinal hormone modulation [51].

Fecal transplantation may reduce the risk of obesity, type 2 diabetes, insulin resistance, and increased BMI [1].

Modulation of intestinal microbiota by using probiotics, prebiotics, antibiotics, and fecal transplantation may have benefits in improving glucose metabolism and insulin resistance [26]. Resveratrol (RSV) is a natural polyphenol with prebiotic benefits found mainly in grapes and berries. Furthermore, 3-dimethyl-1-butanol (DMB) is a structural analogue of choline and an inhibitor of TMA formation by inhibiting microbial enzymes [26, 51].

The interest in the study of resveratrol has started from the fact that the incidence of cardiovascular disease can be decreased by consumption of 150-300 mL/day of red wine. This has led to the extensive use of resveratrol in food supplements with doses ranging from 10-20 mg [26]. Possible mechanisms involve alteration of eicosanoid synthesis, lipid metabolism, and platelet function, as well as inflammatory response, downregulation of proinflammatory mediators, and inhibition of activated immune cells, mainly represented by neutrophils and macrophages. One of the possible mechanisms involves reducing the regulation of inflammatory response by inhibiting the synthesis and release of proinflammatory mediators, modifying the synthesis of eicosanoids and inhibiting activated immune cells [51]. Also, 3,3-dimethyl-1-butanol (DMB) is a choline analogue that inhibits TMA-lyses, a family of bacterial enzymes that transforms multiple substrates into TMA. It is active against the synthesis of TMA not only from choline but also from L-carnitine [52].

Moreover, 3,3-dimethyl-1-butanol is found in certain balsamic vinegars, red wines, and some olive and grape seed oils [46]. DMB promotes microbial taxonomy reduction associated with low plasma levels of TMA and TMAO and has also reduced colonic dietary dependence in developing atherosclerotic lesions [29].

In addition, another drug has been described a few years ago, already known to have cardioprotective clinical effects, by lowering the L-carnitine content in the body [46]. This compound, meldonium (also called Mildronate), has been shown to decrease TMAO by preventing the use of bacterial L-carnitine [52, 53].

## 5. Conclusions

Type 2 diabetes is a complex metabolic disease where concomitant insulin resistance and beta cell damage lead to hyperglycemia. Its proliferation is rapidly and progressively increasing due to an increase in prevalence of obesity and maintaining a western lifestyle in developing countries. Associated complications that come about at some point are related to the major causes of morbidity, mortality, and exceptional healthcare costs. Nowadays, there are no interventional clinical studies showing the beneficial effect of modulating microbiota in CVDs or diabetes.

All available clinical studies are only observational.

Cardiovascular disease (CVD) is a major health problem worldwide. Prospective studies should have demonstrated that patients with diabetes have a two- or four-fold tendency to develop heart failure and acute coronary syndromes, establishing that type 2 DM is an independent risk factor for stroke and heart disease.

In this article, we highlighted aspects relating the involvement of the microbiota and its metabolites to the pathogenesis of diabetes and cardiovascular complications. The profound understanding of the mechanisms involved will allow the early detection of diabetic patients with cardiovascular risk and the formulation of therapeutic regimens in order to reduce the risk of disease progression.
